# Dual antibacterial action of ethyl ferulate as antibiofilm molecule and antibiotic synergist against *Escherichia coli*

**DOI:** 10.3389/fcimb.2026.1789180

**Published:** 2026-04-07

**Authors:** Zhijin Zhang, Yubin Bai, Jing Xu, Rongbin Hu, Zixuan Shang, Xiaojuan Wei, Weiwei Wang, Bing Li, Zhen Zhu, Jiyu Zhang

**Affiliations:** 1 Key Laboratory of New Animal Drug Project of Gansu Province, Key Laboratory of Veterinary Pharmaceutical Development of the Ministry of Agriculture, Lanzhou Institute of Husbandry and Pharmaceutical Sciences of Chinese Academy of Agricultural Sciences (CAAS), Lanzhou, Gansu, China; 2 College of Life Science and Food Engineering, Hebei University of Engineering, Handan, Hebei, China

**Keywords:** antibacterial synergist, antibiofilm, *Escherichia coli*, extracellular polymeric substance, motility

## Abstract

**Introduction:**

The biofilm formed by *Escherichia coli* enhances its pathogenicity and antibiotic resistance, posing a serious threat to human and animal health.

**Methods:**

This study evaluated the anti-biofilm and synergistic antibacterial effects of ethyl ferulate (EF) on *E. coli* using crystal violet (CV) staining and Alamar Blue (AB) assay. The expression levels of biofilm-related genes were analyzed by qRT-PCR.

**Results:**

The results demonstrated that EF effectively inhibited the formation of *E. coli* biofilm and the production of exopolysaccharides (EPS), while enhancing bacterial motility without affecting bacterial growth and metabolic activity. qRT-PCR analysis revealed that EF treatment significantly downregulated the expression of curli-related gene csgD and c-di-GMP-related genes (*pdeR* and *dgcM*), while upregulating the expression of flagella-related genes (*fliC*, *fliM*, and *motB*). Notably, EF exhibited significant antimicrobial synergistic effects when combined with commercially available antibiotics (fosfomycin sodium, cefquinome, gentamicin, tetracycline, and azithromycin), markedly enhancing their antibacterial efficacy.

**Discussion:**

These findings demonstrate that EF possesses potent anti-biofilm activity and holds promise as an antibacterial synergist, offering a promising strategy for the treatment of *E. coli* infections.

## 1 Introduction

*Escherichia coli* is a ubiquitous Gram-negative bacterium that primarily inhabits the intestines of warm-blooded animals and humans, playing a crucial role in maintaining digestive homeostasis and synthesizing vitamin K (Rohatgi and Gupta, 2021; Geng et al., 2022). However, certain strains have evolved into pathogenic Shiga toxin-producing *E. coli* (STEC) through bacteriophage-mediated horizontal gene transfer of virulence plasmids (Newell and La Ragione, 2018). To date, multiple STEC serotypes have been identified, including O26:H11, O91:H21, O111:H8, O157:NM, and the clinically most significant O157:H7 (Bagel et al., 2022). Notably, the O157:H7 serotype has drawn particular attention due to its frequent association with foodborne disease outbreaks and its ability to cause severe complications such as hemorrhagic colitis and hemolytic uremic syndrome (Lim et al., 2010).

Under antibiotic selection pressure, *E. coli* enhances its environmental adaptability through biofilm formation. Biofilms represent structured microbial communities that develop on biotic or abiotic surfaces under favorable environmental conditions, including nutrient availability, appropriate temperature, moisture, and gas exchange (Azeredo et al., 2017).

They are fundamentally structured by extracellular polymeric substances, which consist of exopolysaccharides (EPS), proteins, eDNA, and other components, providing multiple protective mechanisms for bacterial cells (Kaushik et al., 2024).The extracellular polymeric substances can prevent the majority of antibiotics from penetrating into the deeper layers of the biofilm, thereby reducing their efficacy in eradicating the internal bacterial communities (Anderl et al., 2000; Walters et al., 2003; Høiby et al., 2010; Singh et al., 2010). Substantial evidence demonstrates that biofilm-embedded bacteria exhibit significantly enhanced tolerance to disinfectants and antibiotics compared to their planktonic counterparts (Bridier et al., 2015; Giuliano and Rybak, 2015; Williamson et al., 2017). The biofilm formation process is critically dependent on bacterial surface appendages such as fimbriae and flagella (Mitra et al., 2013). Fimbriae, hair-like surface appendages, directly mediate bacterial adhesion to various surfaces (Pratt and Kolter, 1998; Beloin et al., 2004), while flagella, helical filamentous structures protruding from the cell body, are primarily responsible for bacterial motility. Upregulation of key flagellar genes in *E. coli* promotes bacterial motility (Macnab, 1992; Kearns, 2010), maintaining cells in a planktonic state and consequently reducing biofilm formation (Guttenplan and Kearns, 2013).

Given the pivotal role of biofilms in bacterial antibiotic resistance and persistent infections, targeting their formation and maintenance mechanisms has emerged as a crucial research direction in anti-infective therapy. Intervention strategies that inhibit biofilm formation or disrupt their architecture can significantly enhance antibiotic efficacy while reducing the development of drug resistance (Riester et al., 2022; El-Sawy et al., 2024). Consequently, the development of highly effective and low-toxicity biofilm inhibitors, particularly in combination with existing antibiotics, represents a promising innovative therapeutic approach for combating clinically relevant drug-resistant bacterial infections.

Ethyl ferulate (EF) is a natural product widely present in cereal crops such as rice and corn (Cunha et al., 2019). While previous studies have well documented its diverse biological activities, including antioxidant (Xue et al., 2023), anti-inflammatory (Wang et al., 2022), and neuroprotective effects (Scapagnini et al., 2004), research on its anti-biofilm capabilities remains scarce. This study systematically demonstrates the inhibitory effects of EF on *E. coli* biofilms, a preliminary exploration of its potential mechanism of action, and evaluates its synergistic effects with conventional antibiotics.

## 2 Materials and methods

### 2.1 Materials and bacterial strains

The EF used in this study was purchased from MedChemExpress. Based on its water solubility, it was dissolved in dimethyl sulfoxide (DMSO) to prepare a stock solution with a final concentration of 40 mg/mL, ensuring that the concentration of DMSO in the LB medium did not exceed 1%.

*E. coli* O157:H7 (ATCC 43895) was purchased from the American Type Culture Collection(ATCC). Luria-Bertani (LB, HuanKai Microbial, Guangdong, China) and Luria-Bertani agar (LA, HuanKai Microbial, Guangdong, China) medium were used for the growth of *E. coli* strains.

Caco-2 cell lines were obtained from ATCC and cultured under standard conditions using MEM medium with 20% FBS, 1 mM sodium pyruvate, 1 mM L-glutamine, 10 mM HEPES, and 1% non-essential amino acids.

### 2.2 Growth and metabolic activity

The effect of EF on bacterial growth was examined using the microbroth dilution method (CLSI, 2018). Briefly, *E. coli* (10^6^ CFU/mL) was incubated with different concentrations of EF at 37 °C for 24 h. The absorbance at 600 nm was measured after incubation using a microplate reader (Thermo Fisher Scientific, USA).

Meanwhile, the metabolic activity of *E. coli* was analyzed using the Alamar Blue (AB) assay (Swetha et al., 2019). In brief, cells from each well were collected in sterile centrifuge tubes, centrifuged at 5000 rpm for 10 min, washed twice with PBS (pH 7.2), and resuspended in 1 mL of PBS (pH 7.2). Subsequently, the tubes were incubated in the dark at 37 °C for 1 h after adding 10 μL of AB dye (Invitrogen, Thermo Fisher Scientific, USA). The blank control consisted of PBS (pH 7.2) containing only AB dye. The metabolic activity was calculated based on the absorbance at 570 nm and 600 nm using Equation 1. All assays were conducted in three independent replicates.

(1) $$Metabolic\;activity(\%)=\frac{\left((E_{oxi(OD600)}\times T_{OD570})-(E_{oxi(OD570)}T_{OD600})\right)}{\left((E_{red(OD570)}\times B_{OD600})-(E_{red(OD600)}B_{OD570})\right)}\times 100\%$$

Eoxi(OD570)= 80586:Molar extinction coIFAficient of oxidized AB at 570 nm;

Eoxi(OD600)= 117216:Molar extinction coIFAficient of oxidized AB at 600 nm;

Ered(OD570)= 155677:Molar extinction coIFAficient of reduced AB at 570 nm;

Ered(OD600)= 14652:Molar extinction coIFAficient of reduced AB at 600 nm;

### 2.3 Cytotoxicity

The cytotoxicity of EF was evaluated in Caco-2 cells using the CCK-8 assay. Briefly, Caco-2 cells were seeded in 96-well plates at a density of 1 × 10^5^ cells/mL and cultured until the density of the cells reached approximately 80%. The cells were then treated with varying concentrations of EF (3.125, 6.25, 12.5, 25, 50, 100, 200, and 400 µg/mL) for 24 h. Following treatment, 10 μL of CCK-8 reagent (MCE, Shanghai, China) was added to each well, and the plates were incubated for an additional 1 h. Absorbance was measured at 450 nm using a microplate reader. All assays were conducted in three independent replicates.

### 2.4 Biofilm assay

#### 2.4.1 Biofilm inhibition assay

The biofilm quantification was performed using the crystal violet (CV) staining assay according to previously described methods (Batohi et al., 2021). Briefly, *E. coli* was cultured statically at 37 °C for 24 h. The bacterial suspension was then adjusted to an OD_600_ of 0.01 and mixed with various concentrations of EF in a white 96-well plate (Corning Costar^®^ 3599, Corning, Kennebunk, ME, USA). Following 24 h of static incubation at 37 °C, the plates were washed three times with PBS (pH 7.2) to remove non-adherent cells. The biofilms were subsequently fixed with methanol and stained with 0.1% (w/v) CV for 30 min. Unbound dye was removed by rinsing with tap water, and the CV retained in the biofilm was dissolved in 150 μL of 95% ethanol. The absorbance was measured at 595 nm. All assays were conducted in three independent replicates.

#### 2.4.2 Biofilm eradication assay

The biofilm eradication assay was performed according to previously described methods (Shukla and Rao, 2017). Briefly, *E. coli* was cultured statically at 37 °C for 24 h. The bacterial suspension was adjusted to an OD_600_ of 0.01, and 150 μL aliquots were transferred to a 96-well microtiter plate (Corning Costar^®^ 3599, Corning, USA). After 24 h of static incubation at 37 °C to allow biofilm formation, the wells were gently washed three times with phosphate-buffered saline (PBS, pH 7.2). Different concentrations of EF were then added to the pre-formed biofilms, followed by incubation at 37 °C for 24 h. After treatment, the EF-containing medium was carefully aspirated, and the biofilms were washed three times with sterile PBS. The remaining biofilms were fixed with methanol for 30 min and subsequently stained with 0.1% (w/v) crystal violet (CV) for 10 min. Three PBS washes removed unbound dye, and the biofilm-associated CV was solubilized in 150 μL of 95% ethanol. The absorbance was measured at 595 nm using a microplate reader. All assays were conducted in three independent replicates.

#### 2.4.3 Scanning electron microscopy analysis

Observation of bacterial biofilms by SEM was performed according to previously described methods with minor modifications (Yang et al., 2024). Briefly, bacterial suspensions containing different concentrations of EF were inoculated into 96-well plates containing cell culture slides and incubated at 37 °C for 24 h to allow biofilm formation. After incubation, the samples were gently washed three times with sterile PBS to remove planktonic bacteria and loosely adherent cells. The cell culture slides were then carefully extracted from the wells and subjected to sequential fixation and gradient ethanol dehydration. Following gold sputter coating, the prepared samples were examined using an FEI Versa 3D scanning electron microscope (Thermo Fisher Scientific, USA). All assays were conducted in three independent replicates.

### 2.5 EPS production

Quantification of EPS production was performed using the ruthenium red staining method according to previously described protocols (Adnan et al., 2020). Briefly, overnight-cultured *E. coli* was diluted to an OD_600_ of 0.01 in fresh LB broth. The bacterial suspension was then mixed with various concentrations of EF and aliquoted into 96-well plates, followed by static incubation at 37 °C for 24 h to allow EPS production. After three washes with PBS, 200 μL of 0.01% (w/v) ruthenium red solution was added to each well, with a blank control containing only the staining solution. The plates were protected from light and incubated at 37 °C for 60 min. The residual staining solution was subsequently transferred to a fresh 96-well plate, and the absorbance was measured at 450 nm using a microplate reader. The inhibition rate was calculated according to Equation 2. All assays were conducted in three independent replicates.

(2) $$\text{EPS inhibitions}(\%)=\frac{\left(\text{AS}-\text{AP}\right)}{\left(\text{AB}-\text{AP}\right)}\times 100\%$$

AB: Absorbance of the blank control solution, AS: Absorbance of the sample solution, AP: Absorbance of the positive control solution.

### 2.6 Motility (swimming) assay

The effect of EF on *E. coli* motility was evaluated using a previously described method (Vikram et al., 2013). Briefly, overnight cultures of *E. coli* were adjusted to an OD_600_ of 0.01. Semi-solid LB agar (0.3%) plates containing EF at concentrations of 400, 200, and 100 μg/mL were prepared for motility assays. A 1 μL aliquot of the diluted bacterial suspension was inoculated at the center of each plate, followed by incubation at 37 °C for 24 h. Motility was assessed by measuring the diameter of the migration halo zone relative to the control group. All experiments were performed in three independent replicates. All assays were conducted in three independent replicates.

### 2.7 qRT-PCR

The qRT-PCR was performed to investigate the effect of EF on the transcription of biofilm-regulated genes in *E. coli*. *E. coli* was cultured with or without EF for 24 h at 37 °C. Total RNA was extracted using a Bacterial RNA Kit (Omega Bio-tek, Norcross, GA, USA). RNA concentration was measured using a NanoDrop OneC spectrophotometer (Thermo Scientific, USA), and RNA integrity was verified by agarose gel electrophoresis. Subsequently, cDNA was synthesized from RNA using the PrimeScript™ RT Reagent Kit with gDNA Eraser (Takara Bio, Kusatsu, Japan). qRT-PCR was carried out using TB Green^®^ Premix Ex Taq™ II (Tli RNaseH Plus) (Takara Bio, Kusatsu, Japan). Relative gene expression levels were calculated via the 2^−ΔΔCt^ method using a qPCR system (QuantStudio-6Flex machine, Applied Biosystems), with the 16S rRNA gene serving as the internal reference gene. The reaction conditions were set as follows: initial denaturation at 95 °C for 30 minutes, followed by 40 cycles of denaturation at 95 °C for 5 seconds and annealing/extension at 60 °C for 34 seconds. Three biological replicates were set up. The primers used in this study are listed in **Supplementary Table 1**.

### 2.8 Synergistic antibacterial activity of EF combined with antibiotics

The antibacterial efficacy of EF was evaluated according to previously established methods (Liu et al., 2022). Briefly, overnight bacterial cultures were diluted to an OD600 of 0.01 and subsequently mixed with various concentrations of antibiotics (1/2 MIC, 1/4 MIC, and 1/8 MIC) in the presence (400 μg/mL) or absence of EF. The mixtures were then incubated at 37 °C for 16–18 h. Bacterial metabolic activity was subsequently determined using the AB assay. All assays were conducted in three independent replicates.

### 2.9 Statistical analysis

Statistical analysis was performed using GraphPad Prism 9.0 software (San Diego, CA, US). Unless otherwise specified, statistical significance for comparisons was determined by one-way analysis of variance (ANOVA). Prior to conducting ANOVA, the normality of the data and homogeneity of variances were confirmed. *Post hoc* analysis was performed using Dunn’s multiple comparisons test. *P* < 0.05 was considered statistically significant.

## 3 Results

### 3.1 Effects of EF on growth and metabolic activity of *E. coli*

**Figure 1:**
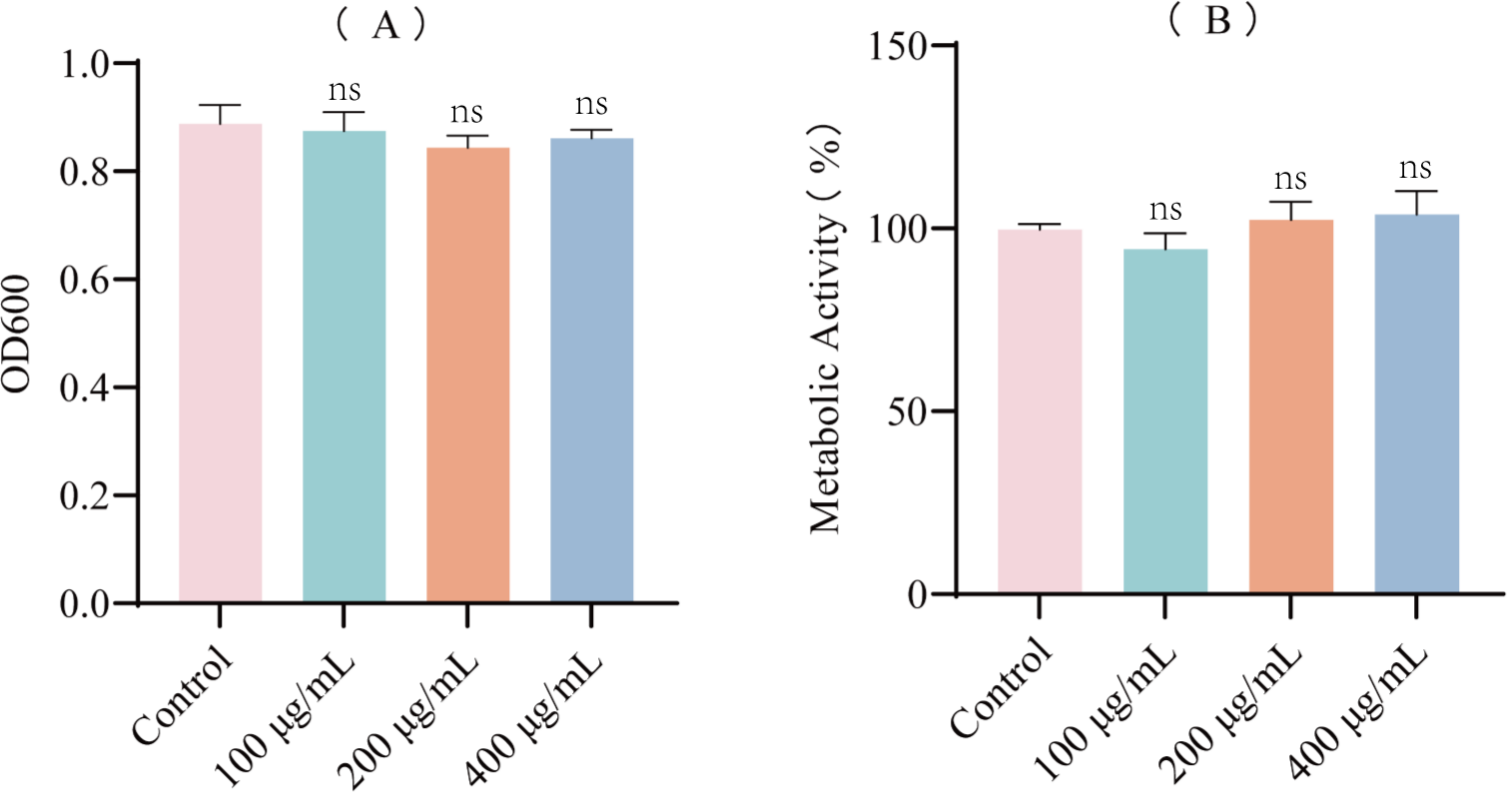
Growth and metabolic activity of *E. coli* in the presence of EF. **(A)** Effect of different concentrations of EF (100, 200, 400 µg/mL) on *E. coli* growth. **(B)** Effect of different concentrations of EF (100, 200, 400 µg/mL) on *E. coli* metabolic activity based on AB assay. All experimental results are presented as mean ± SD of triplicate determinations. Statistical analysis was performed using ANOVA followed by Dunn’s multiple comparisons test. ns: no significant difference compared to the control group.

The effects of EF on the growth and metabolic activity of *E. coli* were evaluated using the microbroth dilution method and AB assay. Compared to the control group, EF at concentrations of 100, 200, and 400 μg/mL showed no significant adverse effects on bacterial growth and metabolic activity (**Figure 1**).

### 3.2 Cytotoxicity of EF on Caco-2 cells

**Figure 2:**
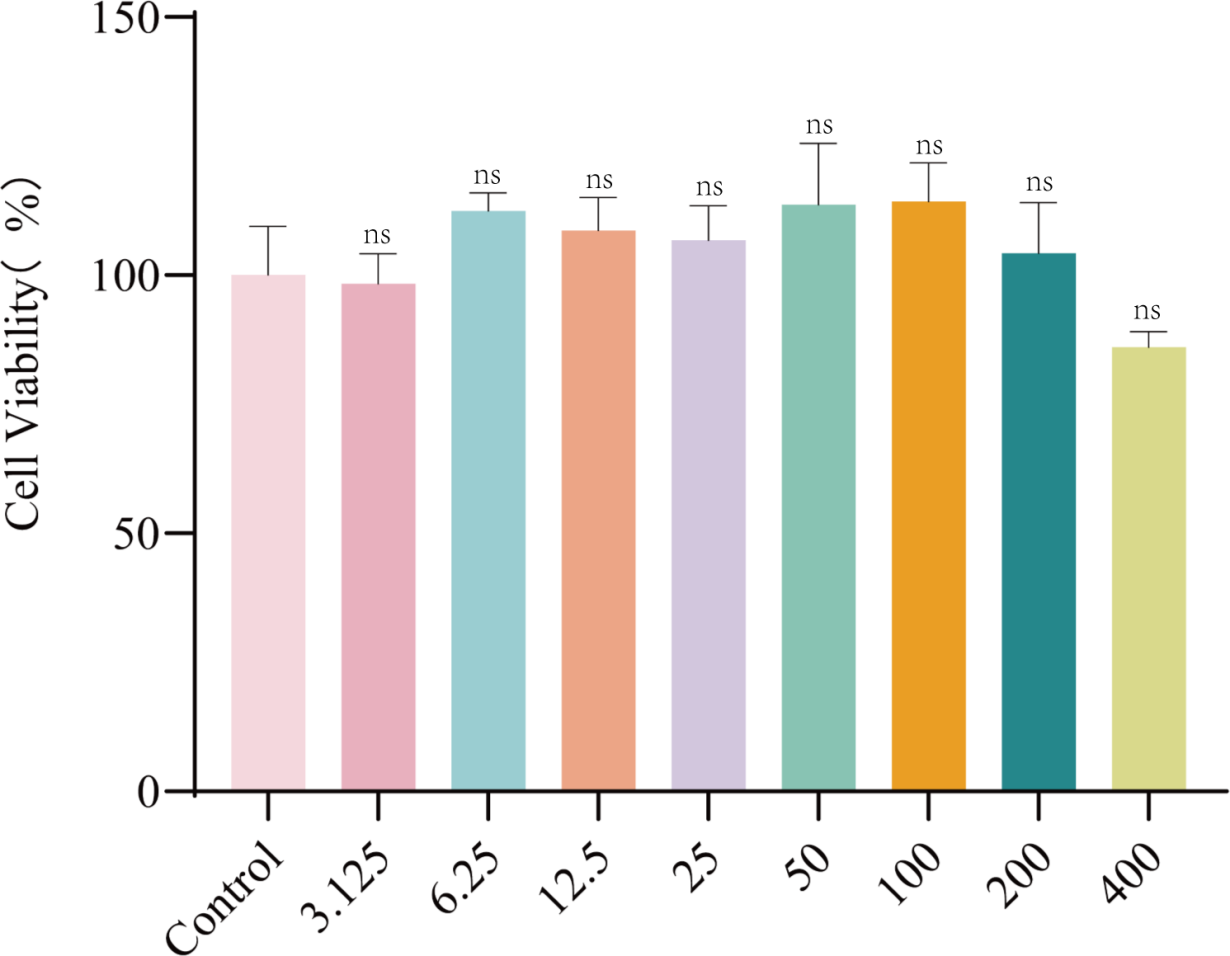
The cytotoxicity of EF in Caco-2 cell. EF was applied to cells at different concentrations (3.125, 6.25, 12.5, 25, 50, 100, 200, and 400 µg/mL) for 24 h. Results from all experiments are presented as the mean ± SD of three replicates. Statistical analysis was performed using Kruskal−Wallis followed by Dunn’s multiple comparisons test. ns: no significant difference compared to the control group.

Next, we evaluated the cytotoxicity of EF toward Caco-2 cells. The results demonstrated that EF exhibited no cytotoxic effects at concentrations of 400 µg/mL or lower(*P*>0.05) (**Figure 2**).

### 3.3 The effect of EF on *E. coli* biofilm

**Figure 3:**
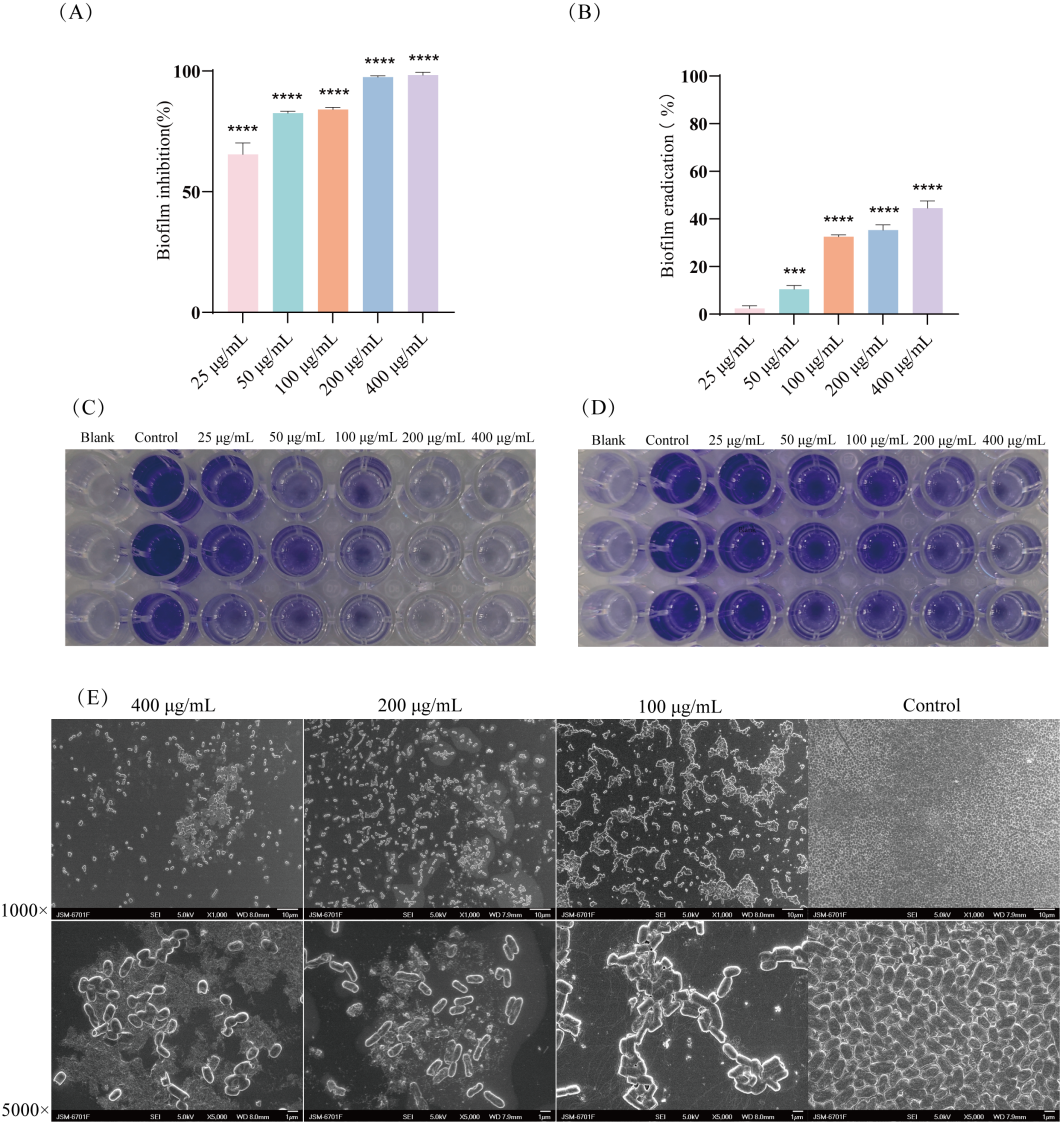
Effects of EF on *E. coli* biofilms. **(A)** and **(C)** Inhibition of biofilm formation by different concentrations of EF (25, 50, 100, 200, and 400 μg/mL) after 24 h treatment. **(B)** and **(D)** Eradication capacity of different EF (25, 50, 100, 200, and 400 μg/mL) against mature biofilms. **(E)** SEM images of *E. coli* biofilms after 24 h treatment with different concentrations of EF (100, 200, and 400 μg/mL). Statistical analysis was performed using Kruskal−Wallis followed by Dunn’s multiple comparisons test. ***=*P* < 0.001, ****=*P* < 0.0001.

Antibiofilm activity of EF was evaluated using CV staining. The biofilm inhibition assay demonstrated that EF treatment significantly suppressed *E. coli* biofilm formation in a dose-dependent manner (**Figures 3A, C**, P < 0.0001). Notably, EF effectively inhibited biofilm formation at concentrations as low as 25 μg/mL (inhibition rate = 65.44%). The biofilm eradication assay further revealed that EF has limited efficacy in eradicating mature biofilms, with a biofilm eradication rate of 42.42% at 400 μg/mL EF (**Figures 3B, D**). To further investigate its antibiofilm effects, SEM imaging analysis was employed. The results confirmed that EF significantly inhibited the formation of biofilms (**Figure 3E**). In conclusion, EF exhibited potent biofilm inhibitory activity, demonstrating significant inhibitory effects even at a low concentration (25 μg/mL); however, its capacity to eradicate preformed biofilms was limited.

### 3.4 Effects of EF on EPS production of *E. coli*

**Figure 4:**
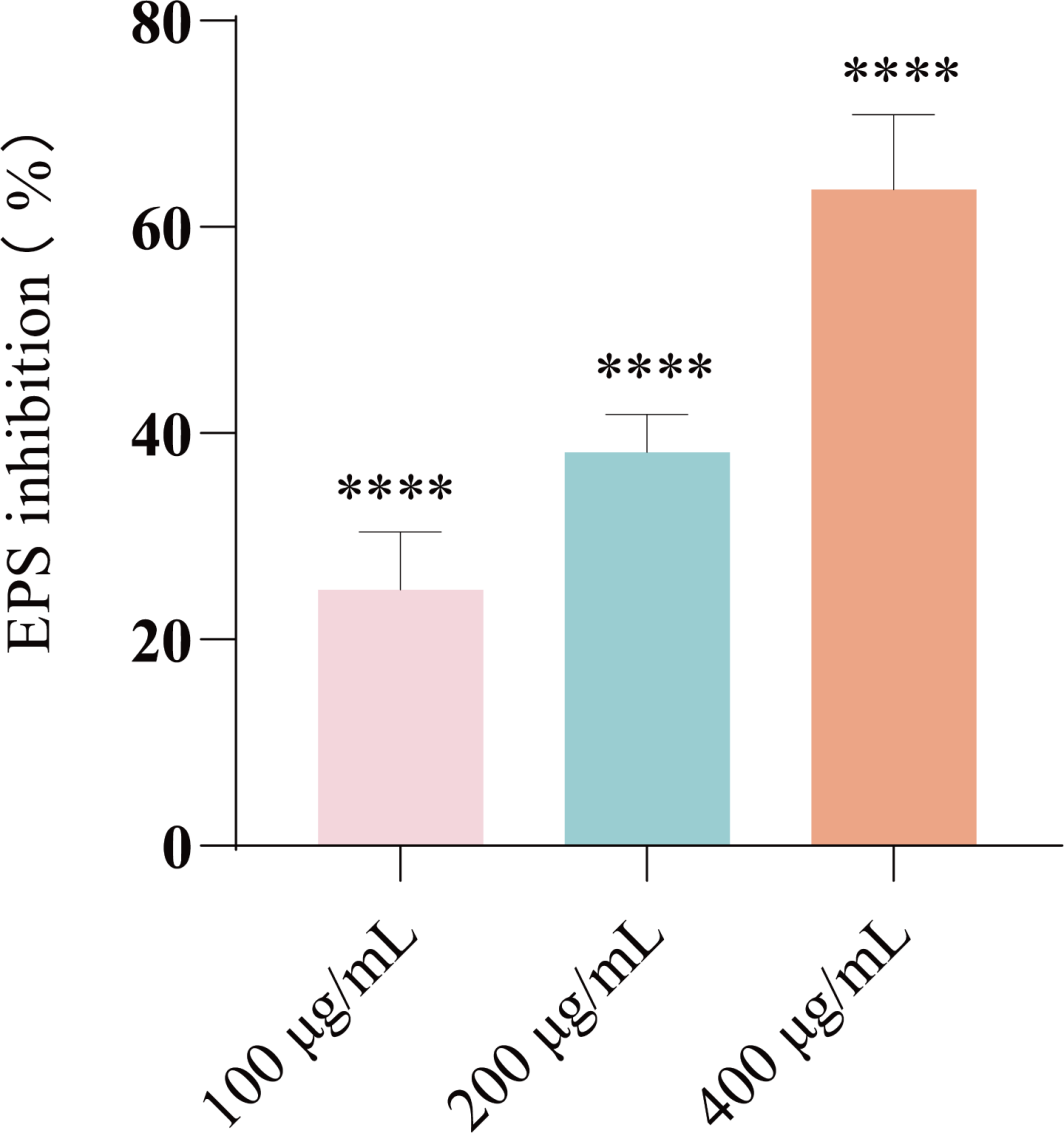
Results of EPS inhibition (%) at various concentrations of EF (100, 200, and 400 μg/mL) for 24 h. Statistical analysis was performed using ANOVA followed by Dunn’s multiple comparisons test. Results from all experiments are presented as the mean ± SD of three replicates. ****P < 0.0001.

The production of EPS in biofilms was investigated using ruthenium red staining. Results demonstrated that EF significantly inhibited EPS production in *E. coli* in a dose-dependent manner (**Figure 4**, *P* < 0.0001). This finding correlates well with the inhibitory effect of EF on *E. coli* biofilm formation.

### 3.5 Effects of EF on the motility of *E. coli*

**Figure 5:**
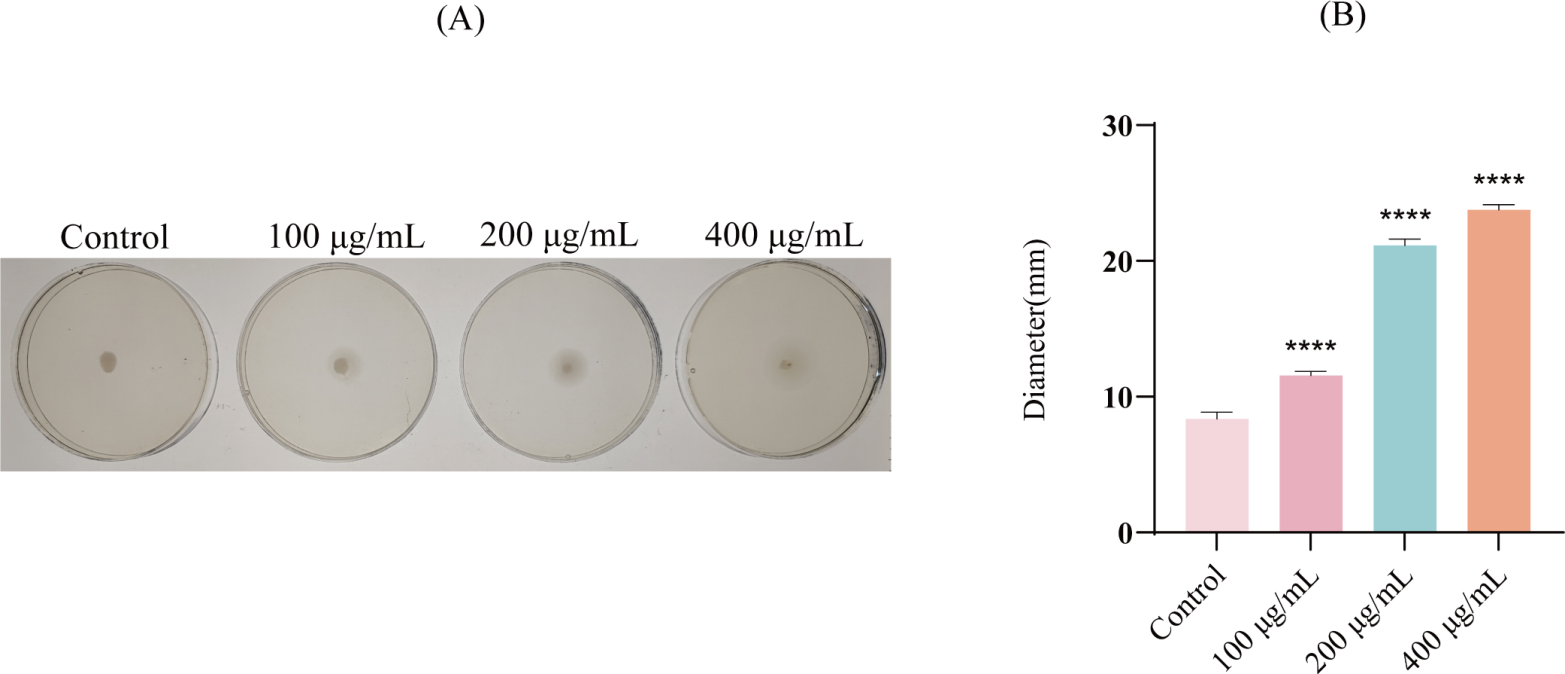
The motility-promoting effect of EF on *E. coli*. **(A)** Motility images of *E. coli* following co-incubation with different concentrations of EF (100, 200, and 400 μg/mL). **(B)** Quantitative assessment of motility based on halo zone diameter. Statistical analysis was performed using ANOVA followed by Dunn’s multiple comparisons test. All experimental results are expressed as mean ± SD of triplicate determinations. ****P < 0.0001.

Biofilm formation is intrinsically linked to bacterial motility. Experimental results demonstrated that EF significantly promoted the motility of *E. coli* in a dose-dependent manner compared to the control group (**Figure 5A**). Furthermore, quantitative analysis of the zone diameters revealed that all EF-treated groups exhibited significantly larger zones than the control (**Figure 5B**, *P* < 0.0001).

### 3.6 Effect of EF on the transcription of biofilm-regulated genes of *E. coli*

**Figure 6:**
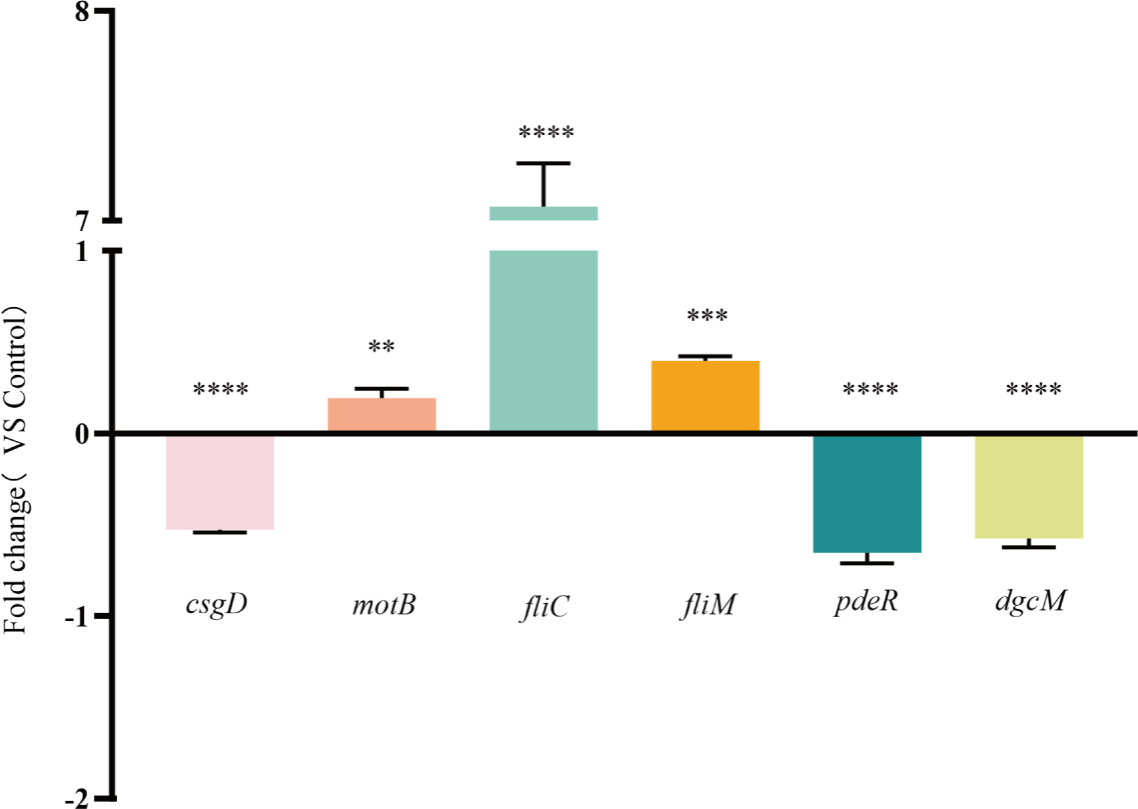
Effects of EF on transcription of biofilm-related genes. qRT-PCR analysis revealed significant transcriptional alterations in six key genes (*csgD*, *motB, fliC*, *fliM*, *pdeR*, and *dgcM*) compared to the control group. Statistical analysis was performed using ANOVA followed by Dunn’s multiple comparisons test. **P < 0.01, ***P < 0.001,****P < 0.0001.

To elucidate the mechanistic basis of EF-mediated biofilm inhibition, we performed qRT-PCR analysis of biofilm-regulated genes, including the curli amyloid gene (*csgD*), flagellar - regulated genes (*fliC*, *fliM*, *motB*), and c-di-GMP - related genes (*pdeR*, *dgcM*). As shown in **Figure 6**, EF treatment exerted differential transcriptional regulation: (i) significant downregulation of *csgD* (52.70% reduction), *pdeR* (65.38% reduction), and *dgcM* (57.40% reduction); (ii) marked upregulation of *fliC* (726.81% increase), *fliM* (39.72% increase), and *motB* (19.37% increase). These results demonstrate that EF disrupts *E. coli* biofilm formation through a dual regulatory mechanism, suppressing curli fiber biosynthesis while enhancing flagellar assembly and motility functions.

### 3.7 Synergistic effects of EF in combination with antibiotics against *E. coli* strains

**Figure 7:**
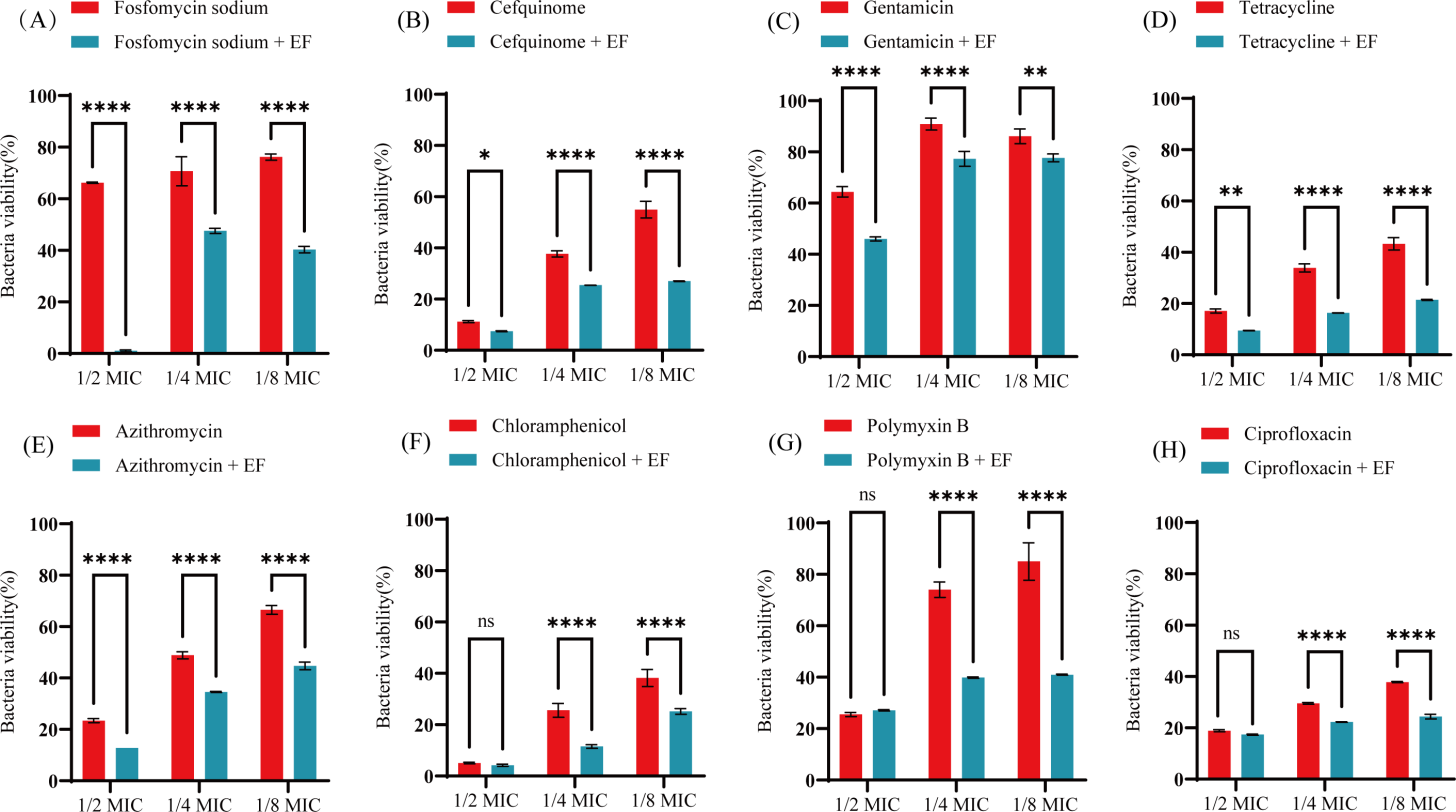
Effects of EF (400 μg/mL) combined with antibiotics on bacterial viability of *E. coli* O157:H7. **(A)** Fosfomycin sodium. **(B)** Cefquinome. **(C)** Gentamicin. **(D)** Tetracycline. **(E)** Azithromycin. **(F)** Chloramphenicol. **(G)** Polymyxin **(B, H)** Ciprofloxacin. Statistical analysis was performed using Kruskal−Wallis followed by Dunn’s multiple comparisons test. All experimental results are presented as mean values ± SD from three independent replicates. ns, no significant difference, *P < 0.05, **P < 0.01, ****P < 0.0001.

Using *E. coli* O157:H7 as a model strain, this study systematically evaluated the synergistic effects between 400 μg/mL EF and sub-inhibitory concentrations of antibiotics (1/2 MIC, 1/4 MIC, and 1/8 MIC) through AB assay. As illustrated in **Figure 7**, EF potentiated the antimicrobial efficacy of fosfomycin sodium, cefquinome, gentamicin, tetracycline, and azithromycin against *E. coli*. Notably, the most pronounced synergistic antibacterial activity was observed when EF was combined with fosfomycin sodium. The concurrent presence of EF and fosfomycin sodium reduced bacterial metabolism by 65.22% compared to fosfomycin sodium alone. These findings suggest that combinatorial therapy with EF and conventional antibiotics may represent a promising therapeutic strategy against biofilm-associated infections caused by *E. coli* and other pathogenic microorganisms.

## 4 Discussion

The formation of biofilms by pathogenic bacteria poses a serious clinical challenge, as it is closely associated with chronic infections and implant failures (Lewis, 2007; Choi et al., 2023). Due to their tolerance to antibiotics and host immune defenses, biofilms often lead to the failure of conventional treatments and increased mortality rates (Muzny and Schwebke, 2015; Zhang et al., 2020; Van Roy and Kielian, 2025). This study is the first to demonstrate the significant inhibitory effect of EF on *E. coli* biofilm formation. Notably, EF effectively inhibited biofilm formation without affecting bacterial growth. This effect may be attributed to the marked suppression of EPS production and the significant enhancement of *E. coli* motility by EF. Furthermore, we demonstrated that EF exhibits synergistic effects when combined with multiple existing antibiotics, including fosfomycin sodium, cefquinome, gentamicin, tetracycline, and azithromycin. Therefore, we conclude that EF exhibits both anti-biofilm and synergistic effects against *E. coli*.

In natural environments and host organisms, the vast majority of bacteria exist as biofilms (Elias and Banin, 2012). Within biofilms, bacteria release functional molecules, including extracellular polymeric substances and autoinducers, to mediate interspecies communication—a critical mechanism for their defense against predation by other species (Rendueles and Ghigo, 2012; Mukherjee and Bassler, 2019). Among these, extracellular polymeric substances, as the key structural components of biofilm formation, not only mediate the firm adhesion of bacteria to surfaces during the initial stage but also promote microbial aggregation (Ghafoor et al., 2011; Harimawan and Ting, 2016; Zhang et al., 2022). The core component of extracellular polymeric substances is EPS, and targeted intervention of EPS may become an effective strategy to disrupt bacterial communication and inhibit biofilm development. Our study demonstrates that EF significantly reduces EPS production in a dose-dependent manner, thereby suppressing biofilm formation. SEM observations further confirmed that EF effectively diminished the biomass of biofilms on cell culture slides. These findings suggest that EF may interfere with biofilm formation by inhibiting EPS production.

The qRT-PCR results in this study demonstrated that EF treatment significantly downregulated the expression of the curli fimbriae-related gene *csgD* and the c-di-GMP-related genes *pdeR* and *dgcM*, while simultaneously upregulating the expression of flagella-related genes *fliC*, *fliM*, and *motB*. The formation and regulation of *E. coli* biofilms involve a complex process mediated by multiple gene expressions, in which *csgD* serves as a core regulatory factor primarily by modulating the synthesis of Curli proteins (Brombacher et al., 2006; Nhu et al., 2018). Studies have shown that aberrant expression of *csgD* disrupts EPS production, thereby impairing bacterial adhesion and the formation of fibrous network structures, ultimately compromising biofilm integrity and environmental adaptability (Yan et al., 2023). EF treatment significantly suppressed *csgD* transcription, inhibiting curli fimbriae production, a finding consistent with EPS quantification data. Furthermore, biofilm formation is dynamically associated with bacterial motility. Research indicates that timely regulation of motility is critical for biofilm development: moderate motility facilitates bacterial surface attachment during the initial phase, whereas timely suppression of motility is essential for stabilizing mature biofilm structures. Sustained high motility impedes the transition of bacterial aggregates into stable biofilm architectures, thereby inhibiting biofilm formation (Guttenplan and Kearns, 2013). Among the motility-related genes, *fliC* encodes the major subunit of flagellar filaments (Chakraborty et al., 2018), *fliM* functions as a component of the C-ring in the flagellar switch complex, regulating directional rotation (Henderson et al., 2020), and *motB* encodes a flagellar motor protein that controls flagellar rotation and motility (Nguyen and Saier, 1996). EF treatment markedly upregulated the transcription of *fliC*, *fliM*, and *motB*, with *fliC* expression increasing by 7.26-fold, indicating that EF significantly enhances *E. coli* motility by promoting flagella-related gene expression, which aligns with motility assay results. In our previous study on Ferulic Acid (FA), an EF-analogous compound, we found that FA inhibits biofilm formation by suppressing the motility of *E. coli*, which is precisely the opposite effect of EF on bacterial motility. This critical difference reveals the distinct anti-biofilm mechanisms between EF and FA, highlighting the unique scientific value of EF. Although FA shares high structural similarity with EF, they exert diametrically opposite regulatory effects on bacterial motility—a key phenotypic trait—suggesting they may act on different molecular targets or signaling pathways. The mode of action of FA aligns with the traditional “anti-colonization” strategy, which prevents biofilm initiation by inhibiting bacterial initial adhesion and motility. In contrast, the effect of EF reveals a novel “anti-maturation” mechanism: by hyperactivating bacterial motility, it may render bacteria unable to successfully transition from the motile, exploratory planktonic state to the stable, aggregated biofilm state, thereby interfering with the maturation and consolidation of biofilm structures. Thus, the study of EF provides a new anti-biofilm strategy complementary to the FA mechanism.

Additionally, EF treatment significantly downregulated the transcription of c-di-GMP-related genes *pdeR* and *dgcM*. As a global regulator, *pdeR* directly or indirectly modulates genes involved in multiple metabolic pathways, and its overexpression promotes EPS production and thicker biofilm formation (Wang et al., 2022). Moreover, *pdeR* inhibits c-di-GMP activity by binding and degrading it, and reduced c-di-GMP activity further enhances bacterial motility (Hengge, 2016). *DgcM*, a c-di-GMP synthase, exhibits decreased expression under EF treatment, leading to lower c-di-GMP levels and consequently increased motility (Serra and Hengge, 2019). Thus, the downregulation of *pdeR* and *dgcM* indirectly enhances bacterial motility by suppressing c-di-GMP activity. In summary, EF likely inhibits biofilm formation through a dual mechanism: (1) by downregulating *csgD* expression to reduce EPS production, directly impairing biofilm formation; and (2) by significantly enhancing bacterial motility, preventing the transition from motile to sessile biofilm-forming states, thereby disrupting biofilm maturation. The synergistic action of these two mechanisms ultimately leads to significant suppression of biofilm formation.

The structured architecture of biofilms confers substantial antibiotic tolerance to bacterial populations, creating a considerable obstacle in clinical treatment (Meroni et al., 2019; Penesyan et al., 2020). Studies have demonstrated that the combined use of antibiotics with biofilm inhibitors can effectively disrupt biofilm structures, thereby markedly increasing bacterial susceptibility to antibiotics (Brackman et al., 2011). Our findings further confirm that anti-biofilm agent EF can substantially enhance the antibacterial efficacy of conventional antibiotics against strains. Regarding the synergistic mechanism between EF and antibiotics, we propose the following two aspects: First, the disruptive effect of EF on the biofilm matrix increases the permeability of antibiotics within the bacterial community, enabling antibiotics to more effectively reach bacterial cells encased within the biofilm. Second, EF may exert a metabolic reactivation effect, restoring sensitivity to antibiotics in bacteria that are in a dormant or low metabolic state. It is particularly noteworthy that EF exhibited a remarkably pronounced synergistic effect when combined with fosfomycin sodium. This phenomenon may be attributed to the unique antibacterial mechanism of fosfomycin sodium, which exerts its effect by inhibiting bacterial cell wall synthesis enzymes. Given that EF significantly upregulates the expression of flagella-related genes (*fliC*, *fliM*, *motB*), this may alter the structure or permeability of the bacterial cell wall, thereby facilitating the entry of fosfomycin sodium into bacterial cells to exert its action. Of course, this hypothesis requires further validation through target binding studies. It should be noted that this study was conducted *in vitro* using a single *E. coli* strain and a static biofilm model. While appropriate for preliminary assessment, this approach cannot fully replicate the dynamic polymicrobial and host microenvironmental conditions *in vivo*. Therefore, further validation using diverse strains, dynamic culture systems, and animal models is essential to comprehensively evaluate the clinical potential of EF. These important discoveries suggest that combination therapy employing antibiotics and anti-biofilm compounds may represent a novel and effective strategy for treating pathogenic infections.

## 5 Conclusions

The findings of this study demonstrate that EF exhibits significant biofilm inhibitory activity and synergistic antibacterial effects against *E. coli*. EF effectively inhibits biofilm formation without affecting normal bacterial growth and metabolic activity; however, its ability to eradicate preformed mature biofilms is limited. The underlying mechanism may be associated with reduced secretion of EPS and enhanced bacterial motility. More importantly, EF shows a marked synergistic antibacterial effect against *E. coli* when combined with fosfomycin sodium, cefquinome, gentamicin, tetracycline, and azithromycin. This potent inhibition coupled with limited eradication suggests that EF is more suitable for combination therapy with conventional antibiotics—enhancing their bactericidal efficacy by suppressing early biofilm formation—rather than for use as a standalone agent against established biofilms. These findings indicate that EF, as a potential biofilm inhibitor, can be used in combination with existing antibiotics, offering a novel strategy for the treatment of biofilm-associated *E. coli* infections.

## Data Availability

The datasets presented in this study can be found in online repositories. The names of the repository/repositories and accession number(s) can be found below: http://doi.org/10.6084/m9.figshare.30860387.
