# Supplementary material for: Dual antibacterial action of ethyl ferulate as antibiofilm molecule and antibiotic synergist against Escherichia coli
Source: Front Cell Infect Microbiol. 2026 Apr 7;16:1789180. doi: 10.3389/fcimb.2026.1789180 (PMC13096051; doi:10.3389/fcimb.2026.1789180)
Supplement: Supplementary file 1 [file Table1.docx]

| Primer Names | **Sequences**（5' to 3'） |
| --- | --- |
| *csgD*-F | AATCGCTGGCAATTACAGG |
| *csgD*-R | CCGCTTCCATCATATCCAG |
| *motB*-F | GTTGTCCCACCGATCCATCTTTCC |
| *motB*-R | CACCAGCACCACCTTCCAGTTC |
| *fliC*-F | TTACCAACCTGAACAACACCACTACC |
| *fliC*-R | ACATATTGGACACTTCGGTCGCATAG |
| *fliM*-F | CCGACCAACCTGAACCTTATCCATC |
| *fliM*-R | CCACGGCGATAAACACCAGACTC |
| *pdeR*-F | CGGCTCCCCTTTCGCATTGG |
| *pdeR*-R | TGTCCTCGTCCGCCTTCCTTC |
| *dgcM-F* | CATCATTTTTCGCCCGATAC |
| *dgcM-R* | CTACCAAGAACCCCACTGGA |
| 16s RNA-left primer 1 | GTGAAGTCATGCCAGGAGCT |
| 16s RNA-right primer 1 | CGAAGTATGCGTCCGGATCA |

**Table S1 Primer sequence information**
